# Supplementary material for: The Small RNA Universe of Capitella teleta
Source: Front Mol Biosci. 2022 Feb 25;9:802814. doi: 10.3389/fmolb.2022.802814 (PMC8915122; doi:10.3389/fmolb.2022.802814)
Supplement: Supplementary file 1 [file DataSheet1.ZIP › Supplement/known/CAPTEscaffold_70_6997.pdf]

| | | |
|---|---|---|
| Provisional ID | : | CAPTEscaffold_70_6997 |
| Score total | : | 4.6 |
| Score for star read(s) | : | -1.3 |
| Score for read counts | : | 0 |
| Score for mfe | : | 1.3 |
| Score for randfold | : | 1.6 |
| Score for cons. seed | : | 3 |
| Total read count | : | 5264456 |
| Mature read count | : | 5264456 |
| Loop read count | : | 0 |
| Star read count | : | 0 |

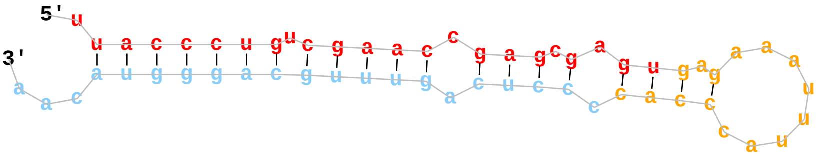


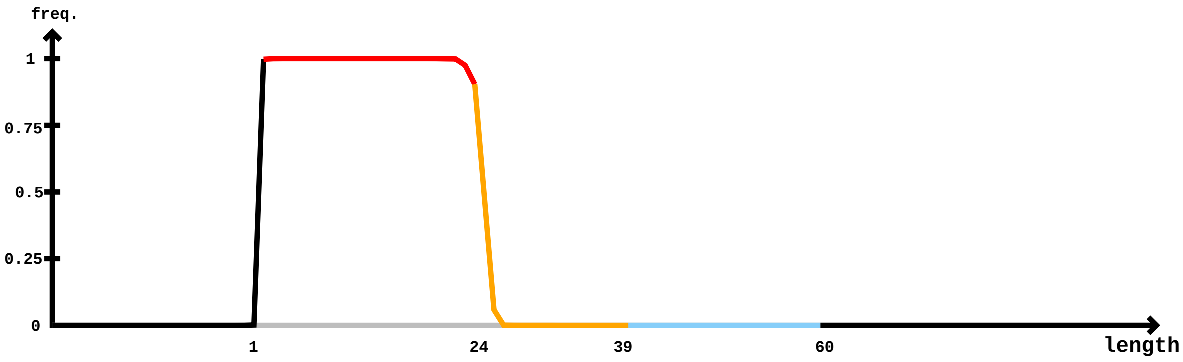


**Mature** **Star**

```
5'- aauuauugguaacguugcccuuacccugucgaaccgagcgagugagaaauuuacccaccccucaguuugcaggguacaagcaacuugcuauuucucaccacgguaugcaguuc   -3'    exp
    aauuauugguaacguugcccuuacccugucgaaccgagcgagugagaaauuuacccaccccucaguuugcaggguacaagcaacuugcuauuucucaccacgguaugcaguuc          known
    ......(((((.(((((...(((((((.(((((.(((.(.(((.(........)))).)))).))))))))))))...)))))))))).........................   reads    mm    sample
    ..................Ucuuacccugucgaaccgagcga........................................................................   20       1     seq
    ..................ccuuacccugucgaaccgagcga........................................................................   3        0     seq
    ..................Acuuacccugucgaaccgagcgag.......................................................................   1        1     seq
    ..................Ucuuacccugucgaaccgagcgag.......................................................................   97       1     seq
    ..................Acuuacccugucgaaccgagcgagu......................................................................   1        1     seq
    ..................ccuuaccUugucgaaccgagcgagu......................................................................   1        1     seq
    ..................Gcuuacccugucgaaccgagcgagu......................................................................   1        1     seq
    ..................Ucuuacccugucgaaccgagcgagu......................................................................   332      1     seq
    ..................ccuuacccugucgaaccAagcgagu......................................................................   1        1     seq
    ..................ccuuacccugucgaaccgagcgagu......................................................................   29       0     seq
    ..................Ucuuacccugucgaaccgagcgagug.....................................................................   2        1     seq
    ...................cuuacccugucgaaccgagc..........................................................................   13       0     seq
    ...................cuuacccugucgaacUgagcga........................................................................   1        1     seq
    ...................cuuacccugucgaaccgagcga........................................................................   152      0     seq
    ...................cuAacccugucgaaccgagcga........................................................................   1        1     seq
    ...................cuuacAcugucgaaccgagcga........................................................................   2        1     seq
    ...................cuuacccugucgaaccAagcga........................................................................   1        1     seq
    ...................cuuacccugucgaaccAagcgag.......................................................................   1        1     seq
    ...................cuuacccugucgaaccgagAgag.......................................................................   1        1     seq
    ...................cuuacccugucgaaccgagcAag.......................................................................   1        1     seq
    ...................cAuacccugucgaaccgagcgag.......................................................................   1        1     seq
    ...................cuuacccugucgUaccgagcgag.......................................................................   1        1     seq
    ...................cuuacccugucgaaccgagcgag.......................................................................   664      0     seq
    ...................cuuacccugucgaGccgagcgag.......................................................................   1        1     seq
    ...................cuuacccugucAaaccgagcgag.......................................................................   2        1     seq
    ...................cuuacccugAcgaaccgagcgag.......................................................................   1        1     seq
    ...................cuuaccUugucgaaccgagcgag.......................................................................   1        1     seq
    ...................cuuacAcugucgaaccgagcgag.......................................................................   3        1     seq
    ...................cuAacccugucgaaccgagcgag.......................................................................   1        1     seq
    ...................cuuacccugucgaUccgagcgag.......................................................................   1        1     seq
    ...................cuuacccugucgaaccgagcgCg.......................................................................   1        1     seq
    ...................cuuacccugucgaaccgGgcgag.......................................................................   1        1     seq
    ...................Auuacccugucgaaccgagcgagu......................................................................   2        1     seq
```

```
                    Mature                      Star
aauuauugguaacguugcccuuacccugucgaaccgagcgagugagaaauuuacccaccccucaguuugcaggguacaagcaacuugcuauuucucaccacgguaugcaguuc
...................cuuacccuguUgaaccgagcgagu......................................................................    1      1    seq
...................cuCacccugucgaaccgagcgagu......................................................................    1      1    seq
...................cuuacccugucgaaUcgagcgagu......................................................................    2      1    seq
...................cuuacGcugucgaaccgagcgagu......................................................................    1      1    seq
...................cuuacccugucgaaccgagcgUgu......................................................................    2      1    seq
...................cuuacccugucgaaccAagcgagu......................................................................    10     1    seq
...................cuuacccugucgaGccgagcgagu......................................................................    1      1    seq
...................cuuGcccugucgaaccgagcgagu......................................................................    1      1    seq
...................cuuacccugucgaaccgUgcgagu......................................................................    1      1    seq
...................cuuacccugucAaaccgagcgagu......................................................................    23     1    seq
...................cuuacccugucgaaccgagcgagA......................................................................    3      1    seq
...................cuuacccugucgaaccgagcgaCu......................................................................    3      1    seq
...................cuuacccuguAgaaccgagcgagu......................................................................    4      1    seq
...................Guuacccugucgaaccgagcgagu......................................................................    4      1    seq
...................cuuacccugucgaaccgagAgagu......................................................................    2      1    seq
...................cuuacccugucgaaccgGgcgagu......................................................................    1      1    seq
...................cuuacccugucgaacAgagcgagu......................................................................    2      1    seq
...................cuuaccUugucgaaccgagcgagu......................................................................    5      1    seq
...................cuuacccuAucgaaccgagcgagu......................................................................    1      1    seq
...................Nuuacccugucgaaccgagcgagu......................................................................    1      1    seq
...................cuuacccugCcgaaccgagcgagu......................................................................    1      1    seq
...................cuuacccugucgaaAcgagcgagu......................................................................    1      1    seq
...................cuuaccAugucgaaccgagcgagu......................................................................    4      1    seq
...................cuuacccAgucgaaccgagcgagu......................................................................    1      1    seq
...................cuuacccGgucgaaccgagcgagu......................................................................    1      1    seq
...................cuuacccugucgaacUgagcgagu......................................................................    3      1    seq
...................cuAacccugucgaaccgagcgagu......................................................................    6      1    seq
...................cuuacccugucgaaccgagcUagu......................................................................    1      1    seq
...................cCuacccugucgaaccgagcgagu......................................................................    1      1    seq
...................cuuacccugAcgaaccgagcgagu......................................................................    1      1    seq
...................cuuacccugucgaaccgagcgNgu......................................................................    1      1    seq
...................cuuacccugucgaaccgagcgagG......................................................................    6      1    seq
...................cuuacccugucgaaccgagcgagu......................................................................    4785   0    seq
...................cuuacccugucgaaccgagcAagu......................................................................    1      1    seq
...................cuuacccugucgaaccUagcgagu......................................................................    1      1    seq
...................cNuacccugucgaaccgagcgagu......................................................................    1      1    seq
...................Uuuacccugucgaaccgagcgagu......................................................................    13     1    seq
...................cuuaccGugucgaaccgagcgagu......................................................................    1      1    seq
...................cuuacAcugucgaaccgagcgagu......................................................................    17     1    seq
...................cuuacccugucgaaccgagcgaguU.....................................................................    241    1    seq
...................Auuacccugucgaaccgagcgagug.....................................................................    13     1    seq
...................cuuacccugucgaaccgagcgagugU....................................................................    28     1    seq
...................cuuacccugucgaaccgagcgagugG....................................................................    3      1    seq
....................uuacccugucgaacAgag...........................................................................    1      1    seq
....................Auacccugucgaaccgag...........................................................................    3      1    seq
....................uuacccugucgaaccgaA...........................................................................    9      1    seq
....................uuacAcugucgaaccgag...........................................................................    2      1    seq
....................uuacccugAcgaaccgag...........................................................................    1      1    seq
....................uuacccAgucgaaccgag...........................................................................    1      1    seq
....................uuacccugucAaaccgag...........................................................................    2      1    seq
....................uuacccugucgUaccgag...........................................................................    1      1    seq
....................uuacccugucgaacUgag...........................................................................    1      1    seq
....................uuacccugucgaaccAag...........................................................................    1      1    seq
....................uuacccugucgaaccgGg...........................................................................    1      1    seq
....................uGacccugucgaaccgag...........................................................................    1      1    seq
....................uuacccugucgaaccgag...........................................................................    682    0    seq
....................uuacccugucgaGccgagc..........................................................................    1      1    seq
....................uuacccugAcgaaccgagc..........................................................................    3      1    seq
....................uuacUcugucgaaccgagc..........................................................................    1      1    seq
....................uuacccugucgaaccgagA..........................................................................    194    1    seq
....................uuacccugucAaaccgagc..........................................................................    7      1    seq
....................uAacccugucgaaccgagc..........................................................................    1      1    seq
....................uuaccAugucgaaccgagc..........................................................................    2      1    seq
....................Guacccugucgaaccgagc..........................................................................    2      1    seq
....................uuacccugucgaacAgagc..........................................................................    1      1    seq
....................uuacccugucgaaccgagG..........................................................................    4      1    seq
....................uuacccugucgGaccgagc..........................................................................    1      1    seq
....................uuacccuCucgaaccgagc..........................................................................    1      1    seq
....................Nuacccugucgaaccgagc..........................................................................    1      1    seq
....................uuacccugucgaaccgGgc..........................................................................    1      1    seq
```

**Mature** **Star**

```
aauuauugguaacguugcccuuacccugucgaaccgagcgagugagaaauuuacccaccccucaguuugcagguacaagcaacuugcuauuucucaccacgguaugcaguuc
....................uuacccugucgaaccgUgc.........................................................................   1   1   seq
....................uuacccuguAgaaccgagc.........................................................................   1   1   seq
....................uuacccuguUgaaccgagc.........................................................................   2   1   seq
....................Auacccugucgaaccgagc.........................................................................   14   1   seq
....................uuacccAgucgaaccgagc.........................................................................   1   1   seq
....................uuacccCgucgaaccgagc.........................................................................   3   1   seq
....................uuacccugucgaacUgagc.........................................................................   2   1   seq
....................uuacccugucgaaccgagU.........................................................................   6   1   seq
....................uuacccugucgaaccgagc.........................................................................   2865   0   seq
....................uuGcccugucgaaccgagc.........................................................................   1   1   seq
....................uuacccugucgaaccAagc.........................................................................   2   1   seq
....................uuacccuAucgaaccgagc.........................................................................   1   1   seq
....................uuacAcugucgaaccgagc.........................................................................   3   1   seq
....................uuacccugucgaaccAagcg........................................................................   2   1   seq
....................uuacccugucgaaccgagAg........................................................................   3   1   seq
....................uCacccugucgaaccgagcg........................................................................   1   1   seq
....................uuacccuAucgaaccgagcg........................................................................   1   1   seq
....................uuacccugucAaaccgagcg........................................................................   5   1   seq
....................uuacccCgucgaaccgagcg........................................................................   1   1   seq
....................Guacccugucgaaccgagcg........................................................................   1   1   seq
....................uAacccugucgaaccgagcg........................................................................   1   1   seq
....................uuacccuguUgaaccgagcg........................................................................   1   1   seq
....................uuacccugucgaaccgagcA........................................................................   1   1   seq
....................uuacccuguAgaaccgagcg........................................................................   1   1   seq
....................uuacccAgucgaaccgagcg........................................................................   1   1   seq
....................uuCcccugucgaaccgagcg........................................................................   1   1   seq
....................uuacccugucgaaccgagcU........................................................................   6   1   seq
....................Nuacccugucgaaccgagcg........................................................................   1   1   seq
....................uuaccAugucgaaccgagcg........................................................................   2   1   seq
....................Auacccugucgaaccgagcg........................................................................   6   1   seq
....................uuacccugucgaaccgagcg........................................................................   2276   0   seq
....................uuaGccugucgaaccgagcg........................................................................   1   1   seq
....................uuacccugucgaaAcgagcg........................................................................   1   1   seq
....................uuacccugucgaaccgagUg........................................................................   1   1   seq
....................uuacccugucgCaccgagcga.......................................................................   4   1   seq
....................uuacccugucgaaccAagcga.......................................................................   121   1   seq
....................uuaccGugucgaaccgagcga.......................................................................   6   1   seq
....................uuacccugucgaaccgagcUa.......................................................................   11   1   seq
....................uNacccugucgaaccgagcga.......................................................................   4   1   seq
....................uuacGcugucgaaccgagcga.......................................................................   5   1   seq
....................uuacccugucgaaccgagGga.......................................................................   6   1   seq
....................uCacccugucgaaccgagcga.......................................................................   13   1   seq
....................uuacccugucgUaccgagcga.......................................................................   13   1   seq
....................uuacccugucgaaccgagUga.......................................................................   20   1   seq
....................uuacccugucUaaccgagcga.......................................................................   4   1   seq
....................uuacccuAucgaaccgagcga.......................................................................   16   1   seq
....................uuGcccugucgaaccgagcga.......................................................................   17   1   seq
....................uuacccuNucgaaccgagcga.......................................................................   1   1   seq
....................Auacccugucgaaccgagcga.......................................................................   459   1   seq
....................uuacccugucgaGccgagcga.......................................................................   27   1   seq
....................uuacccugucgaaccgagAga.......................................................................   35   1   seq
....................uuacccugucgaacGgagcga.......................................................................   5   1   seq
....................uuaccAugucgaaccgagcga.......................................................................   73   1   seq
....................uuacccugucgaUccgagcga.......................................................................   11   1   seq
....................uuacccugucgaaUcgagcga.......................................................................   14   1   seq
....................uuacccugucgaaccgagcgU.......................................................................   333   1   seq
....................uuacccuguUgaaccgagcga.......................................................................   86   1   seq
....................uuacccugucCaaccgagcga.......................................................................   7   1   seq
....................uuacccugucgaaccgGgcga.......................................................................   20   1   seq
....................uuacccuCucgaaccgagcga.......................................................................   19   1   seq
....................uuacccuUucgaaccgagcga.......................................................................   4   1   seq
....................Nuacccugucgaaccgagcga.......................................................................   27   1   seq
....................uuacccugCcgaaccgagcga.......................................................................   22   1   seq
....................uuacccugucgaaccgCgcga.......................................................................   2   1   seq
....................uuacccugucgaaccgagcga.......................................................................   122263   0   seq
....................Cuacccugucgaaccgagcga.......................................................................   11   1   seq
....................uuacccugAcgaaccgagcga.......................................................................   42   1   seq
....................uuacccugucgaaccgagcAa.......................................................................   37   1   seq
....................uuaccNugucgaaccgagcga.......................................................................   2   1   seq
....................uuacccuguAgaaccgagcga.......................................................................   51   1   seq
```

```
                    Mature                            Star
aauuauugguaacguugcccuuacccugucgaaccgagcgagugagaaauuuacccaccccucaguuugcagguacaagcaacuugcuauuucucaccacgguaugcaguuc
....................uuacccugucgaaccgagcgN.......................................................................    2     1    seq
....................uuacccuguGgaaccgagcga.......................................................................    5     1    seq
....................uuacccugucgaaAcgagcga.......................................................................    17    1    seq
....................uuacccCgucgaaccgagcga.......................................................................    15    1    seq
....................uAacccugucgaaccgagcga.......................................................................    79    1    seq
....................uuUcccugucgaaccgagcga.......................................................................    5     1    seq
....................uuacccugucgaaccgaAcga.......................................................................    7     1    seq
....................uuaAccugucgaaccgagcga.......................................................................    22    1    seq
....................uuacccGgucgaaccgagcga.......................................................................    4     1    seq
....................uuaUccugucgaaccgagcga.......................................................................    11    1    seq
....................uGacccugucgaaccgagcga.......................................................................    2     1    seq
....................uuacccugucgaacUgagcga.......................................................................    59    1    seq
....................uuacccugucgGaccgagcga.......................................................................    15    1    seq
....................Guacccugucgaaccgagcga.......................................................................    43    1    seq
....................uuacccugucgaaccUagcga.......................................................................    7     1    seq
....................uuaccUugucgaaccgagcga.......................................................................    63    1    seq
....................uuacccugucgaaccgagcgG.......................................................................    359   1    seq
....................uuacccugucgaCccgagcga.......................................................................    2     1    seq
....................uuacccugucgaaccgaUcga.......................................................................    4     1    seq
....................uuacccugucgaaccgagcCa.......................................................................    5     1    seq
....................uuacAcugucgaaccgagcga.......................................................................    97    1    seq
....................uuacUcugucgaaccgagcga.......................................................................    31    1    seq
....................uuacccAgucgaaccgagcga.......................................................................    72    1    seq
....................uuacccugucgaacAgagcga.......................................................................    39    1    seq
....................uuacccugucgaaGcgagcga.......................................................................    2     1    seq
....................uuacccugGcgaaccgagcga.......................................................................    10    1    seq
....................uuacccugucgaaccgUgcga.......................................................................    4     1    seq
....................uuacccugucgaaccgaCcga.......................................................................    1     1    seq
....................uuacccugucAaaccgagcga.......................................................................    310   1    seq
....................uuacccugucgaaccCagcga.......................................................................    9     1    seq
....................uuGcccugucgaaccgagcgag......................................................................    72    1    seq
....................Nuacccugucgaaccgagcgag......................................................................    84    1    seq
....................uNacccugucgaaccgagcgag......................................................................    7     1    seq
....................uuacccugucgUaccgagcgag......................................................................    36    1    seq
....................uuacccugucgaaccCagcgag......................................................................    11    1    seq
....................uuacccugucNaaccgagcgag......................................................................    1     1    seq
....................uuacccugucgaaccgagcAag......................................................................    211   1    seq
....................uuacccugucgaaccAagcgag......................................................................    440   1    seq
....................uuacccugucgaaccgagcgNg......................................................................    3     1    seq
....................uuacccuguGgaaccgagcgag......................................................................    26    1    seq
....................uuaUccugucgaaccgagcgag......................................................................    26    1    seq
....................uuacccugucgaaccgagcgUg......................................................................    44    1    seq
....................uuaAccugucgaaccgagcgag......................................................................    81    1    seq
....................uAacccugucgaaccgagcgag......................................................................    241   1    seq
....................uGacccugucgaaccgagcgag......................................................................    3     1    seq
....................uuacccugAcgaaccgagcgag......................................................................    123   1    seq
....................uuacccugucgGaccgagcgag......................................................................    36    1    seq
....................uuacccuguUgaaccgagcgag......................................................................    130   1    seq
....................uuacccugucgaaccgagcgGg......................................................................    338   1    seq
....................uuacccugucAaaccgagcgag......................................................................    841   1    seq
....................uuacccugucgaaccgagcgaC......................................................................    36    1    seq
....................uuacccuAucgaaccgagcgag......................................................................    60    1    seq
....................uuacccugucgaaccgagcgaN......................................................................    3     1    seq
....................uuacccugucgaacUgagcgag......................................................................    146   1    seq
....................uuacccugucgaaccgGgcgag......................................................................    55    1    seq
....................uuacccugucgaaGcgagcgag......................................................................    9     1    seq
....................uuacccugucgaaccgUgcgag......................................................................    28    1    seq
....................uuacccugucgaUccgagcgag......................................................................    28    1    seq
....................uuacccugCcgaaccgagcgag......................................................................    60    1    seq
....................uuacccugGcgaaccgagcgag......................................................................    16    1    seq
....................uuaccAugucgaaccgagcgag......................................................................    227   1    seq
....................uuacccuCucgaaccgagcgag......................................................................    58    1    seq
....................uuacccAgucgaaccgagcgag......................................................................    175   1    seq
....................uuacccugucgaaccgaCcgag......................................................................    15    1    seq
....................uuacccGgucgaaccgagcgag......................................................................    17    1    seq
....................Auacccugucgaaccgagcgag......................................................................    1182  1    seq
....................uuacccugucgaaccgagcgaA......................................................................    1128  1    seq
....................uuacccugucgaaUcgagcgag......................................................................    29    1    seq
....................uuacccugucgaaccgagcgaU......................................................................    815   1    seq
....................uuacccugucgaaccgagcUag......................................................................    40    1    seq
```

```
                    Mature                                    Star
aauuauugguaacguugcccuuacccugucgaaccgagcgagugagaaauuuacccaccccucaguuugcagguacaagcaacuugcuauuucucaccacgguaugcaguuc
....................uuacccuguAgaaccgagcgag......................................................................  152     1  seq
....................Cuacccugucgaaccgagcgag......................................................................  32      1  seq
....................uuacccugucgaaccgaUcgag......................................................................  17      1  seq
....................uuaccNugucgaaccgagcgag......................................................................  15      1  seq
....................Guacccugucgaaccgagcgag......................................................................  169     1  seq
....................uuacccugucgaacGgagcgag......................................................................  13      1  seq
....................uuacccuUucgaaccgagcgag......................................................................  91      1  seq
....................uuacccugucgaaccUagcgag......................................................................  41      1  seq
....................uuacccugucgaaccgagAgag......................................................................  134     1  seq
....................uuaccGugucgaaccgagcgag......................................................................  13      1  seq
....................uuacccugucgaaccgagcgag......................................................................  364493  0  seq
....................uuacccugucUaaccgagcgag......................................................................  22      1  seq
....................uuaGccugucgaaccgagcgag......................................................................  13      1  seq
....................uuacccCgucgaaccgagcgag......................................................................  33      1  seq
....................uuacccugucgaGccgagcgag......................................................................  76      1  seq
....................uuUcccugucgaaccgagcgag......................................................................  31      1  seq
....................uuacccugucgCaccgagcgag......................................................................  4       1  seq
....................uuacAcugucgaaccgagcgag......................................................................  285     1  seq
....................uuacccugucCaaccgagcgag......................................................................  20      1  seq
....................uuacccuguNgaaccgagcgag......................................................................  1       1  seq
....................uuacccugucgaaccgagcgCg......................................................................  11      1  seq
....................uuacccugucgaaccgaAcgag......................................................................  14      1  seq
....................uuacccugucgaaccgagcCag......................................................................  12      1  seq
....................uuacccugucgaaccgagGgag......................................................................  28      1  seq
....................uuacccugucgaCccgagcgag......................................................................  2       1  seq
....................uuCcccugucgaaccgagcgag......................................................................  11      1  seq
....................uuacccugucgaaNcgagcgag......................................................................  1       1  seq
....................uuacccugucgaaAcgagcgag......................................................................  33      1  seq
....................uuacccugucgaacAgagcgag......................................................................  106     1  seq
....................uuNcccugucgaaccgagcgag......................................................................  2       1  seq
....................uuacccugucgaaccgagUgag......................................................................  203     1  seq
....................uuacUcugucgaaccgagcgag......................................................................  92      1  seq
....................uuacccugNcgaaccgagcgag......................................................................  2       1  seq
....................uCacccugucgaaccgagcgag......................................................................  67      1  seq
....................uuacGcugucgaaccgagcgag......................................................................  17      1  seq
....................uuaccUugucgaaccgagcgag......................................................................  199     1  seq
....................uuacccugucgaaccgCgcgag......................................................................  5       1  seq
....................uuacccugucgaaccgagcgagC.....................................................................  442     1  seq
....................uuacccugucgaaccgagGgagu.....................................................................  269     1  seq
....................uuNcccugucgaaccgagcgagu.....................................................................  17      1  seq
....................uuacccugucgaaccgagUgagu.....................................................................  595     1  seq
....................uuacccugucgaCccgagcgagu.....................................................................  17      1  seq
....................uGacccugucgaaccgagcgagu.....................................................................  69      1  seq
....................uuGcccugucgaaccgagcgagu.....................................................................  612     1  seq
....................uCacccugucgaaccgagcgagu.....................................................................  1004    1  seq
....................uuacccugucgaaccgGgcgagu.....................................................................  669     1  seq
....................Auacccugucgaaccgagcgagu.....................................................................  14429   1  seq
....................uuacAcugucgaaccgagcgagu.....................................................................  2930    1  seq
....................uuacccugucgaaccgagcUagu.....................................................................  357     1  seq
....................uuacccugucgaaccgagcAagu.....................................................................  3532    1  seq
....................uuacccugucgaaccgUgcgagu.....................................................................  323     1  seq
....................uuCcccugucgaaccgagcgagu.....................................................................  42      1  seq
....................uuacccugucgaaccCagcgagu.....................................................................  186     1  seq
....................uuacccugucgaGccgagcgagu.....................................................................  970     1  seq
....................uuacccugucgaaccgagcgagA.....................................................................  42018   1  seq
....................uuacccugucgaaccAagcgagu.....................................................................  7491    1  seq
....................uuacccugucgaaccgagcgNgu.....................................................................  31      1  seq
....................Nuacccugucgaaccgagcgagu.....................................................................  1038    1  seq
....................uuacccuUucgaaccgagcgagu.....................................................................  273     1  seq
....................uuacccuguUgaaccgagcgagu.....................................................................  1878    1  seq
....................uuaGccugucgaaccgagcgagu.....................................................................  155     1  seq
....................uuacccugucgaaccgaCcgagu.....................................................................  137     1  seq
....................uuacccugucgaaccgNgcgagu.....................................................................  14      1  seq
....................uuacccugucgaaNcgagcgagu.....................................................................  12      1  seq
....................uuacccugucgaaccgagcgagG.....................................................................  6139    1  seq
....................uuacccugucgaaccgaAcgagu.....................................................................  278     1  seq
....................uuacccuCucgaaccgagcgagu.....................................................................  593     1  seq
....................uuacccugucgUaccgagcgagu.....................................................................  395     1  seq
....................uuacccugucgaaccgaNcgagu.....................................................................  1       1  seq
....................uuUcccugucgaaccgagcgagu.....................................................................  210     1  seq
```

```
                    Mature                          Star
aauuauugguaacguugcccuuacccugucgaaccgagcgagugagaaauuuacccaccccucaguuugcagguacaagcaacuugcuauuucucaccacgguaugcaguuc
....................uuacccugucgaaUcgagcgagu.....................................................................	365	1	seq
....................uuacccugucgNaccgagcgagu.....................................................................	3	1	seq
....................uuacccugucgaaccgagcgGgu.....................................................................	511	1	seq
....................uuacccugucgCaccgagcgagu.....................................................................	56	1	seq
....................uuacGcugucgaaccgagcgagu.....................................................................	203	1	seq
....................uuacccugucNaaccgagcgagu.....................................................................	8	1	seq
....................uuacccugucgaaGcgagcgagu.....................................................................	91	1	seq
....................uuacccugucgaaccgagcCagu.....................................................................	145	1	seq
....................uuacccuguGgaaccgagcgagu.....................................................................	287	1	seq
....................uuacccugucgaaccgagcgCgu.....................................................................	27	1	seq
....................uuaccGugucgaaccgagcgagu.....................................................................	116	1	seq
....................uuacccugucgaaccgagcgaAu.....................................................................	245	1	seq
....................uuaUccugucgaaccgagcgagu.....................................................................	356	1	seq
....................uuacccugucgaaAcgagcgagu.....................................................................	467	1	seq
....................uuacccugucgaaccgagcgaCu.....................................................................	180	1	seq
....................uuacccugucgaaccgagAgagu.....................................................................	1356	1	seq
....................uuacccugucgaaccgagcgaUu.....................................................................	225	1	seq
....................uuacNcugucgaaccgagcgagu.....................................................................	1	1	seq
....................uuacccugucUaaccgagcgagu.....................................................................	303	1	seq
....................uNacccugucgaaccgagcgagu.....................................................................	134	1	seq
....................uuacccugucgaaccNagcgagu.....................................................................	9	1	seq
....................uuacccugGcgaaccgagcgagu.....................................................................	227	1	seq
....................uuacccuAucgaaccgagcgagu.....................................................................	656	1	seq
....................uuacccugucgaaccgagcNagu.....................................................................	7	1	seq
....................uuacccugucgaaccUagcgagu.....................................................................	364	1	seq
....................uuacccugCcgaaccgagcgagu.....................................................................	620	1	seq
....................uuacccAgucgaaccgagcgagu.....................................................................	1944	1	seq
....................uuacccugucgaaccgagcgUgu.....................................................................	432	1	seq
....................uuaccNugucgaaccgagcgagu.....................................................................	98	1	seq
....................uuacccugucgaaccgagcgagu.....................................................................	4144500	0	seq
....................uuacccugucgaaccgagcgaNu.....................................................................	11	1	seq
....................Guacccugucgaaccgagcgagu.....................................................................	1936	1	seq
....................uAacccugucgaaccgagcgagu.....................................................................	2581	1	seq
....................uuacccuguAgaaccgagcgagu.....................................................................	1914	1	seq
....................uuacUcugucgaaccgagcgagu.....................................................................	1317	1	seq
....................uuacccugucAaaccgagcgagu.....................................................................	14454	1	seq
....................uuacccugucgaacAgagcgagu.....................................................................	1191	1	seq
....................uuaccUugucgaaccgagcgagu.....................................................................	2433	1	seq
....................uuacccugucgaacUgagcgagu.....................................................................	2007	1	seq
....................uuaAccugucgaaccgagcgagu.....................................................................	769	1	seq
....................uuacccugNcgaaccgagcgagu.....................................................................	2	1	seq
....................uuacccugucgaaccgCgcgagu.....................................................................	90	1	seq
....................uuacccugucgaUccgagcgagu.....................................................................	301	1	seq
....................uuacccugucCaaccgagcgagu.....................................................................	189	1	seq
....................uuacccGgucgaaccgagcgagu.....................................................................	189	1	seq
....................uuacccugucgaacGgagcgagu.....................................................................	116	1	seq
....................uuacccugucgGaccgagcgagu.....................................................................	442	1	seq
....................uuacccugucgaaccgagcgagN.....................................................................	84	1	seq
....................uuacccuNucgaaccgagcgagu.....................................................................	4	1	seq
....................uuacccugAcgaaccgagcgagu.....................................................................	1365	1	seq
....................Cuacccugucgaaccgagcgagu.....................................................................	375	1	seq
....................uuacccugucgaaccgaUcgagu.....................................................................	266	1	seq
....................uuacccuguNgaaccgagcgagu.....................................................................	34	1	seq
....................uuacccCgucgaaccgagcgagu.....................................................................	682	1	seq
....................uuacccugucgaaccgagNgagu.....................................................................	6	1	seq
....................uuacccNgucgaaccgagcgagu.....................................................................	2	1	seq
....................uuaccAugucgaaccgagcgagu.....................................................................	2326	1	seq
....................uuacUcugucgaaccgagcgagug....................................................................	24	1	seq
....................uuacccugucgaaccgagcgaguC....................................................................	30	1	seq
....................uuacccugucgaaccgagGgagug....................................................................	8	1	seq
....................uuacccugucgaGccgagcgagug....................................................................	27	1	seq
....................uGacccugucgaaccgagcgagug....................................................................	2	1	seq
....................uuaccNugucgaaccgagcgagug....................................................................	4	1	seq
....................uuacccugucgaaccgagcgaAug....................................................................	3	1	seq
....................uuacccuguUgaaccgagcgagug....................................................................	35	1	seq
....................uNacccugucgaaccgagcgagug....................................................................	4	1	seq
....................uuacccugucgaaccgagcgUgug....................................................................	13	1	seq
....................uuacccugucgCaccgagcgagug....................................................................	1	1	seq
....................uuacccugucgaaccgagUgagug....................................................................	5	1	seq
....................uuacccugucgaaccgagcCagug....................................................................	6	1	seq
```

**Mature** **Star**

| | | | |
|---|---|---|---|
| aauuauugguaacguugcccuuacccugucgaaccgagcgagugagaaauuuacccaccccucaguuugcaggguacaagcaacuugcuauuucucaccacgguaugcaguuc | | | |
| ....................uuacccugucAaaccgagcgagug..................................................................... | 256 | 1 | seq |
| ....................uuacccugucgGaccgagcgagug..................................................................... | 8 | 1 | seq |
| ....................uuacccugucgaaccgagcgaguA..................................................................... | 1782 | 1 | seq |
| ....................uuaccAugucgaaccgagcgagug..................................................................... | 49 | 1 | seq |
| ....................uuacccugucgaaccUagcgagug..................................................................... | 9 | 1 | seq |
| ....................uuacccugucgaaccCagcgagug..................................................................... | 3 | 1 | seq |
| ....................uuacccuUucgaaccgagcgagug..................................................................... | 5 | 1 | seq |
| ....................uuacccCgucgaaccgagcgagug..................................................................... | 10 | 1 | seq |
| ....................uuacccugucgaaccgCgcgagug..................................................................... | 1 | 1 | seq |
| ....................Auacccugucgaaccgagcgagug..................................................................... | 261 | 1 | seq |
| ....................uuacccugucgaaccgaCcgagug..................................................................... | 1 | 1 | seq |
| ....................uuacccugucgaaccgUgcgagug..................................................................... | 7 | 1 | seq |
| ....................uuacccugucgaaccgagAgagug..................................................................... | 34 | 1 | seq |
| ....................uuacccuguAgaaccgagcgagug..................................................................... | 107 | 1 | seq |
| ....................uuacccugCcgaaccgagcgagug..................................................................... | 14 | 1 | seq |
| ....................uuaGccugucgaaccgagcgagug..................................................................... | 1 | 1 | seq |
| ....................uuacccugucgaaGcgagcgagug..................................................................... | 2 | 1 | seq |
| ....................uuaAccugucgaaccgagcgagug..................................................................... | 20 | 1 | seq |
| ....................uuacccugucgaaAcgagcgagug..................................................................... | 4 | 1 | seq |
| ....................uuacccugAcgaaccgagcgagug..................................................................... | 30 | 1 | seq |
| ....................uuacccugucgaaccgagcgNgug..................................................................... | 1 | 1 | seq |
| ....................uuacccugucgaacGgagcgagug..................................................................... | 3 | 1 | seq |
| ....................uuacccugucgaaccgagcgaCug..................................................................... | 5 | 1 | seq |
| ....................uuacccugucgaaccgagcUagug..................................................................... | 10 | 1 | seq |
| ....................uuacccugucgaaUcgagcgagug..................................................................... | 4 | 1 | seq |
| ....................uCacccugucgaaccgagcgagug..................................................................... | 18 | 1 | seq |
| ....................Guacccugucgaaccgagcgagug..................................................................... | 33 | 1 | seq |
| ....................uuacccuAucgaaccgagcgagug..................................................................... | 11 | 1 | seq |
| ....................uuaUccugucgaaccgagcgagug..................................................................... | 6 | 1 | seq |
| ....................uuacccugucgaacUgagcgagug..................................................................... | 40 | 1 | seq |
| ....................uuacccugucgaaccgagcgGgug..................................................................... | 10 | 1 | seq |
| ....................uuacccugucgaaccgagcgagGg..................................................................... | 46 | 1 | seq |
| ....................uuUcccugucgaaccgagcgagug..................................................................... | 3 | 1 | seq |
| ....................uuacccugucgaaccgagcgagAg..................................................................... | 30 | 1 | seq |
| ....................uuacccugucgaaccgagcgaUug..................................................................... | 2 | 1 | seq |
| ....................Cuacccugucgaaccgagcgagug..................................................................... | 4 | 1 | seq |
| ....................uuacccugucUaaccgagcgagug..................................................................... | 5 | 1 | seq |
| ....................uuacccuguNgaaccgagcgagug..................................................................... | 1 | 1 | seq |
| ....................uuacccugucgaUccgagcgagug..................................................................... | 11 | 1 | seq |
| ....................uuacccuguGgaaccgagcgagug..................................................................... | 3 | 1 | seq |
| ....................uuacccugucgaaccgaUcgagug..................................................................... | 5 | 1 | seq |
| ....................uuacccugucgaaccgagcgagug..................................................................... | 77420 | 0 | seq |
| ....................uuacccugucCaaccgagcgagug..................................................................... | 5 | 1 | seq |
| ....................uuacccugucgaaccgGgcgagug..................................................................... | 14 | 1 | seq |
| ....................uuacccAgucgaaccgagcgagug..................................................................... | 34 | 1 | seq |
| ....................uuacAcugucgaaccgagcgagug..................................................................... | 47 | 1 | seq |
| ....................uuacccugucgaaccgagcgaguN..................................................................... | 4 | 1 | seq |
| ....................uuacccugucgaaccgagcgagCg..................................................................... | 12 | 1 | seq |
| ....................uuacccugucgaaccAagcgagug..................................................................... | 139 | 1 | seq |
| ....................uuacccugucgaacAgagcgagug..................................................................... | 20 | 1 | seq |
| ....................uuaccUugucgaaccgagcgagug..................................................................... | 51 | 1 | seq |
| ....................Nuacccugucgaaccgagcgagug..................................................................... | 25 | 1 | seq |
| ....................uuaccGugucgaaccgagcgagug..................................................................... | 1 | 1 | seq |
| ....................uuacccugGcgaaccgagcgagug..................................................................... | 5 | 1 | seq |
| ....................uuacccugucgaaccgaAcgagug..................................................................... | 3 | 1 | seq |
| ....................uuGcccugucgaaccgagcgagug..................................................................... | 9 | 1 | seq |
| ....................uuacccugucgaaccgagcgaguU..................................................................... | 79695 | 1 | seq |
| ....................uuacccugucgaaccgagcAagug..................................................................... | 62 | 1 | seq |
| ....................uAacccugucgaaccgagcgagug..................................................................... | 49 | 1 | seq |
| ....................uuCcccugucgaaccgagcgagug..................................................................... | 2 | 1 | seq |
| ....................uuacccuCucgaaccgagcgagug..................................................................... | 13 | 1 | seq |
| ....................uuacccugucgUaccgagcgagug..................................................................... | 5 | 1 | seq |
| ....................uuacccugucgUaccgagcgaguga.................................................................... | 6 | 1 | seq |
| ....................uuacccuAucgaaccgagcgaguga.................................................................... | 4 | 1 | seq |
| ....................uuacccugucgaaccCagcgaguga.................................................................... | 2 | 1 | seq |
| ....................uuacccugucgaaUcgagcgaguga.................................................................... | 1 | 1 | seq |
| ....................uuacccugucgaaccgGgcgaguga.................................................................... | 4 | 1 | seq |
| ....................uuacccugucgaaccgagcgaguUa.................................................................... | 325 | 1 | seq |
| ....................uuUcccugucgaaccgagcgaguga.................................................................... | 1 | 1 | seq |
| ....................uuacccuguAgaaccgagcgaguga.................................................................... | 11 | 1 | seq |

```
                    Mature                       Star
aauuauugguaacguugcccuuacccugucgaaccgagcgagugagaaauuuacccaccccucaguuugcaggguacaagcaacuugcuauuucucaccacgguaugcaguuc
....................uuacccugucgaaccgagUgaguga....................................................................    3        1    seq
....................uuacccugucgaaccgagcgagugN....................................................................    4        1    seq
....................uuacccugucgaaccgagcgagugC....................................................................    1049     1    seq
....................uuacccugucgaaccgagcgaguga....................................................................    30396    0    seq
....................uuacccugucgaaccgagcgagNga....................................................................    3        1    seq
....................uuaccNugucgaaccgagcgaguga....................................................................    1        1    seq
....................uuacccAgucgaaccgagcgaguga....................................................................    19       1    seq
....................uNacccugucgaaccgagcgaguga....................................................................    1        1    seq
....................uuacccugucgaaccgagcgGguga....................................................................    3        1    seq
....................uuacccugucgaaccgagcgagCga....................................................................    5        1    seq
....................Nuacccugucgaaccgagcgaguga....................................................................    8        1    seq
....................uuacccugucgaaccgaAcgaguga....................................................................    1        1    seq
....................uuacccugucgaacAgagcgaguga....................................................................    11       1    seq
....................Auacccugucgaaccgagcgaguga....................................................................    114      1    seq
....................uuacccugucCaaccgagcgaguga....................................................................    1        1    seq
....................uuaccUugucgaaccgagcgaguga....................................................................    14       1    seq
....................uuaAccugucgaaccgagcgaguga....................................................................    5        1    seq
....................uuacccugucgaaccgagcgaguCa....................................................................    1        1    seq
....................uuacccugucgGaccgagcgaguga....................................................................    2        1    seq
....................uuaUccugucgaaccgagcgaguga....................................................................    5        1    seq
....................uuacccugucgaaccgagAgaguga....................................................................    9        1    seq
....................uuacccugucgaaGcgagcgaguga....................................................................    1        1    seq
....................uuaccGugucgaaccgagcgaguga....................................................................    1        1    seq
....................uuaGccugucgaaccgagcgaguga....................................................................    1        1    seq
....................uuacccugucgaacGgagcgaguga....................................................................    1        1    seq
....................uuacccugucgaaccgagcUaguga....................................................................    1        1    seq
....................uuacccugucgaaccgagcgUguga....................................................................    4        1    seq
....................uuacccugGcgaaccgagcgaguga....................................................................    1        1    seq
....................uuacccugucgaaccAagcgaguga....................................................................    30       1    seq
....................uuacccCgucgaaccgagcgaguga....................................................................    1        1    seq
....................uuacccugAcgaaccgagcgaguga....................................................................    17       1    seq
....................uuGcccugucgaaccgagcgaguga....................................................................    4        1    seq
....................uuacccGgucgaaccgagcgaguga....................................................................    1        1    seq
....................Guacccugucgaaccgagcgaguga....................................................................    19       1    seq
....................uuacccugucgaaccgagcgagAga....................................................................    8        1    seq
....................uuacccuCucgaaccgagcgaguga....................................................................    6        1    seq
....................uuacccugucgaaccgagcgagugU....................................................................    252254   1    seq
....................uuCcccugucgaaccgagcgaguga....................................................................    2        1    seq
....................uuacAcugucgaaccgagcgaguga....................................................................    27       1    seq
....................uuacccugucgaaccgUgcgaguga....................................................................    2        1    seq
....................uuacccuguUgaaccgagcgaguga....................................................................    13       1    seq
....................uuacUcugucgaaccgagcgaguga....................................................................    12       1    seq
....................uuacccuguGgaaccgagcgaguga....................................................................    1        1    seq
....................uuaccAugucgaaccgagcgaguga....................................................................    15       1    seq
....................uuacccuUucgaaccgagcgaguga....................................................................    3        1    seq
....................Cuacccugucgaaccgagcgaguga....................................................................    2        1    seq
....................uuacccugucgaaccgagcgaguAa....................................................................    230      1    seq
....................uCacccugucgaaccgagcgaguga....................................................................    6        1    seq
....................uuacccugucgaacUgagcgaguga....................................................................    9        1    seq
....................uuacccugucgaaccgagcAaguga....................................................................    22       1    seq
....................uuacccugucgaUccgagcgaguga....................................................................    4        1    seq
....................uuacccugucgaaccgaUcgaguga....................................................................    2        1    seq
....................uuacGcugucgaaccgagcgaguga....................................................................    3        1    seq
....................uAacccugucgaaccgagcgaguga....................................................................    20       1    seq
....................uuacccugucgaaccgagcgaUuga....................................................................    2        1    seq
....................uuacccugucgaaccgagcgagugG....................................................................    16869    1    seq
....................uuacccugucUaaccgagcgaguga....................................................................    1        1    seq
....................uuacccugCcgaaccgagcgaguga....................................................................    3        1    seq
....................uuacccugucgaaccgagcgaAuga....................................................................    2        1    seq
....................uuacccugucAaaccgagcgaguga....................................................................    62       1    seq
....................uuacccugucgaaccgagGgaguga....................................................................    4        1    seq
....................uuacccugucgaGccgagcgaguga....................................................................    7        1    seq
....................uuacccugucgaaAcgagcgaguga....................................................................    6        1    seq
....................uuacccugucgaaccgagcgagugaC...................................................................    1        1    seq
....................uuacccugucgaaccgagcgagugaU...................................................................    2280     1    seq
....................uuacccugucgaaccgagcgagugaA...................................................................    499      1    seq
....................uuacccugucgaaccgagcgagugUg...................................................................    41       1    seq
....................uuacccugucgaaccgagcgagugaUa..................................................................    1        1    seq
....................uuacccugucgaaccgagcgagugaCa..................................................................    6        1    seq
....................uuacccugucgaaccgagcgagugaAa..................................................................    318      1    seq
```

```
                    Mature                                    Star
aauuauugguaacguugcccuuacccugucgaaccgagcgagugagaaauuuacccaccccucaguuugcagguacaagcaacuugcuauuucucaccacgguaugcaguuc
....................uuacccugucgaaccgagcgagugaAaa................................................................    83      1    seq
....................uuacccugucgaaccgagcgagugaAaaa...............................................................    79      1    seq
....................Auacccugucgaaccgagcgagugagaaauu.............................................................    2       1    seq
....................uAacccugucgaaccgagcgagugagaaauu.............................................................    1       1    seq
....................uuacccugucgaGccgagcgagugagaaauu.............................................................    1       1    seq
....................uuacccugucgaaccgaCcgagugagaaauu.............................................................    1       1    seq
....................uuacccugucgaaccgagcgagugagaaauu.............................................................    356     0    seq
....................uuacccugucgaaccgagAgagugagaaauu.............................................................    1       1    seq
....................uuacccugucgaaccAagcgagugagaaauu.............................................................    1       1    seq
....................uuUcccugucgaaccgagcgagugagaaauu.............................................................    1       1    seq
....................uuacccugucgaaAcgagcgagugagaaauu.............................................................    1       1    seq
....................uuacccugucgaaccgagcgagugagaaaAu.............................................................    1       1    seq
.....................uacccuguAgaaccgagc.........................................................................    7       1    seq
.....................uUcccugucgaaccgagcg........................................................................    1       1    seq
.....................uacccugucgaaccgagcga.......................................................................    72      0    seq
.....................uaAccugucgaaccgagcga.......................................................................    2       1    seq
.....................uacAcugucgaaccgagcga.......................................................................    1       1    seq
.....................uacccugucgaaccgagcgG.......................................................................    1       1    seq
.....................uUcccugucgaaccgagcga.......................................................................    6       1    seq
.....................uacccugucAaaccgagcga.......................................................................    1       1    seq
.....................uacccGgucgaaccgagcgag......................................................................    2       1    seq
.....................uacccugucgaaccgagcgag......................................................................    629     0    seq
.....................Aacccugucgaaccgagcgag......................................................................    6       1    seq
.....................Gacccugucgaaccgagcgag......................................................................    2       1    seq
.....................uacccugucgaUccgagcgag......................................................................    1       1    seq
.....................uacccAgucgaaccgagcgag......................................................................    1       1    seq
.....................uacccugucgaacAgagcgag......................................................................    1       1    seq
.....................uUcccugucgaaccgagcgag......................................................................    26      1    seq
.....................uacccugucAaaccgagcgag......................................................................    2       1    seq
.....................uacccugucgaaUcgagcgagu.....................................................................    1       1    seq
.....................uacccugucgaUccgagcgagu.....................................................................    1       1    seq
.....................Gacccugucgaaccgagcgagu.....................................................................    103     1    seq
.....................Nacccugucgaaccgagcgagu.....................................................................    2       1    seq
.....................uacccugucgCaccgagcgagu.....................................................................    1       1    seq
.....................uacccugucgaaAcgagcgagu.....................................................................    2       1    seq
.....................uacUcugucgaaccgagcgagu.....................................................................    1       1    seq
.....................uacccugucgaaccgagcgaAu.....................................................................    1       1    seq
.....................uacccugucgaaccgagUgagu.....................................................................    2       1    seq
.....................uacccCgucgaaccgagcgagu.....................................................................    2       1    seq
.....................uaAccugucgaaccgagcgagu.....................................................................    5       1    seq
.....................uaccUugucgaaccgagcgagu.....................................................................    8       1    seq
.....................uacccuCucgaaccgagcgagu.....................................................................    1       1    seq
.....................uaccGugucgaaccgagcgagu.....................................................................    2       1    seq
.....................uacccugucgaaccgagcgagu.....................................................................    7516    0    seq
.....................uacccugucgaaccgagcgGgu.....................................................................    2       1    seq
.....................uacccugucgaGccgagcgagu.....................................................................    2       1    seq
.....................uacccugucgaacAgagcgagu.....................................................................    1       1    seq
.....................uacccuguAgaaccgagcgagu.....................................................................    13      1    seq
.....................uacccugucgaaccgagcUagu.....................................................................    2       1    seq
.....................uacccugCcgaaccgagcgagu.....................................................................    3       1    seq
.....................uacccugucgaaccgagcgagC.....................................................................    2       1    seq
.....................uacccuguUgaaccgagcgagu.....................................................................    3       1    seq
.....................uacGcugucgaaccgagcgagu.....................................................................    1       1    seq
.....................uacccugucgaacGgagcgagu.....................................................................    1       1    seq
.....................uacccuUucgaaccgagcgagu.....................................................................    1       1    seq
.....................uacccGgucgaaccgagcgagu.....................................................................    5       1    seq
.....................uacccugucgaaccgagcAagu.....................................................................    5       1    seq
.....................uacccugucgaaccgagcgaUu.....................................................................    1       1    seq
.....................uacccugucgaaccAagcgagu.....................................................................    11      1    seq
.....................uUcccugucgaaccgagcgagu.....................................................................    129     1    seq
.....................uacAcugucgaaccgagcgagu.....................................................................    9       1    seq
.....................uacccugucAaaccgagcgagu.....................................................................    16      1    seq
.....................uacccugucgaacUgagcgagu.....................................................................    7       1    seq
.....................Aacccugucgaaccgagcgagu.....................................................................    39      1    seq
.....................uacccuAucgaaccgagcgagu.....................................................................    2       1    seq
.....................uacccugucgaaccgagAgagu.....................................................................    2       1    seq
.....................uacccugucgaaccgagcgagG.....................................................................    14      1    seq
.....................uCcccugucgaaccgagcgagu.....................................................................    1       1    seq
.....................uacccAgucgaaccgagcgagu.....................................................................    3       1    seq
.....................uacccugucUaaccgagcgagu.....................................................................    1       1    seq
```

```
                    Mature                                Star
aauuauugguaacguugcccuuacccugucgaaccgagcgagugagaaauuuacccaccccucaguuugcagguacaagcaacuugcuauuucucaccacgguaugcaguuc
.....................uacccugucgaaccgagcgagA.....................................................................    92     1     seq
.....................Cacccugucgaaccgagcgagu.....................................................................    1      1     seq
.....................uacccugucgaaUcgagcgagug....................................................................    1      1     seq
.....................uUcccugucgaaccgagcgagug....................................................................    3      1     seq
.....................Aacccugucgaaccgagcgagug....................................................................    1      1     seq
.....................uacccugucgaaccgagcgagug....................................................................    192    0     seq
.....................uacccugucgaaccgagcgaguU....................................................................    485    1     seq
.....................uacccugucgaaccgagcgaguUa...................................................................    1      1     seq
.....................uacccugucgaaccgagcgaguga...................................................................    90     0     seq
.....................uUcccugucgaaccgagcgaguga...................................................................    1      1     seq
.....................uacccugucgaaccgagcgagugU...................................................................    686    1     seq
.....................uacccugucgaaccgagcgagugG...................................................................    53     1     seq
.....................uacccugucgaaccgagcgagugaU..................................................................    17     1     seq
.....................uacccugucgaaccgagcgagugaga.................................................................    1      0     seq
......................acccugucgaaccgagcga.......................................................................    27     0     seq
......................Gcccugucgaaccgagcga.......................................................................    2      1     seq
......................acccuguAgaaccgagcga.......................................................................    1      1     seq
......................acccAgucgaaccgagcga.......................................................................    15     1     seq
......................acccAgucgaaccgagcgag......................................................................    14     1     seq
......................acccugucgaaccgagcgag......................................................................    46     0     seq
......................Ccccugucgaaccgagcgag......................................................................    1      1     seq
......................acccugucgaaccgagcAagu.....................................................................    1      1     seq
......................acccugucgaaccgagcgagG.....................................................................    2      1     seq
......................Gcccugucgaaccgagcgagu.....................................................................    18     1     seq
......................acccugucgaaccAagcgagu.....................................................................    1      1     seq
......................acccugucgaacUgagcgagu.....................................................................    1      1     seq
......................acccugucgaaccgagcgagA.....................................................................    17     1     seq
......................Ucccugucgaaccgagcgagu.....................................................................    3      1     seq
......................acccuguGgaaccgagcgagu.....................................................................    1      1     seq
......................acccugucgaaAcgagcgagu.....................................................................    1      1     seq
......................acccugucgaaccgagcgagu.....................................................................    740    0     seq
......................acccuguAgaaccgagcgagu.....................................................................    7      1     seq
......................acccAgucgaaccgagcgagu.....................................................................    219    1     seq
......................aAccugucgaaccgagcgagu.....................................................................    1      1     seq
......................acccCgucgaaccgagcgagu.....................................................................    2      1     seq
......................acccugucgaaccgagcgaguU....................................................................    13     1     seq
......................acccugucgaaccgagcgagug....................................................................    3      0     seq
......................acccugucgaaccgagcgagugU...................................................................    120    1     seq
......................acccugucgaaccgagcgagugG...................................................................    9      1     seq
......................acccugucgaaccgagcgaguga...................................................................    7      0     seq
......................acccAgucgaaccgagcgaguga...................................................................    2      1     seq
......................acccugucgaaccgagcgagugaU..................................................................    10     1     seq
.......................cccugucAaaccgagcgagu.....................................................................    1      1     seq
.......................cccAgucgaaccgagcgagu.....................................................................    1      1     seq
.......................cccugucgaaccgagcgagu.....................................................................    31     0     seq
........................ccugucgaaccgagcgagu.....................................................................    6      0     seq
.........................cugucgaaccgagcgagu.....................................................................    11     0     seq
```
